# A qualitative study on determinants of the use of malaria rapid diagnostic test and anti-malarial drug prescription practices by primary healthcare workers in Ebonyi state, Nigeria

**DOI:** 10.1186/s12936-024-04958-3

**Published:** 2024-04-25

**Authors:** Ugwu I. Omale

**Affiliations:** Department of Community Medicine, Alex Ekwueme Federal University Teaching Hospital Abakaliki (AEFUTHA), Abakaliki, Ebonyi State Nigeria

**Keywords:** Use, Malaria RDT, Anti-malarial drug prescription practices, Primary healthcare workers, Determinants, Qualitative, Nigeria

## Abstract

**Background:**

The increased availability and use of malaria rapid diagnostic test (RDT) by primary healthcare (PHC) workers has made universal diagnostic testing before malaria treatment more feasible. However, to meaningfully resolve the problem of over-treatment with artemisinin-based combination therapy and the heightened risk of selection pressure and drug resistance, there should be appropriate response (non-prescription of anti-malarial drugs) following a negative RDT result by PHC workers. This study explored the determinants of the use of RDT and anti-malarial drug prescription practices by PHC workers in Ebonyi state, Nigeria.

**Methods:**

Between March 2 and 10, 2020, three focus group discussions were conducted in English with 23 purposively-selected consenting PHC workers involved in the diagnosis and treatment of malaria. Data was analysed thematically as informed by the method by Braun and Clarke.

**Results:**

The determinants of the use of RDT for malaria diagnosis were systemic (RDT availability and patient load), provider related (confidence in RDT and the desire to make correct diagnosis, PHC worker’s knowledge and training, and fear to prick a patient), client related (fear of needle prick and refusal to receive RDT, and self-diagnosis of malaria, based on symptoms, and insistence on not receiving RDT), and RDT-related (the ease of conducting and interpreting RDT). The determinants of anti-malarial drug prescription practices were systemic (drug availability and cost) and drug related (effectiveness and side-effects of the drugs). The determinants of the prescription of anti-malarial drugs following negative RDT were provider related (the desire to make more money and limited confidence in RDT) and clients’ demand while unnecessary co-prescription of antibiotics with anti-malarial drugs following positive RDT was determined by the desire to make more money.

**Conclusions:**

This evidence highlights many systemic, provider, client, and RDT/drug related determinants of PHC workers’ use of RDT and anti-malarial drug prescription practices that should provide tailored guidance for relevant health policy actions in Ebonyi state, Nigeria, and similar settings.

## Background

Malaria is a preventable and curable disease but it continues to take a toll on populations across the world, especially children under 5 years old and pregnant women in Nigeria and other high burden countries [1, 2]. About 27% of the global malaria cases and 31% of the global malaria deaths in 2021 occurred in Nigeria which has the highest burden of malaria in the world [1] and Ebonyi state has one of the highest burden of malaria in Nigeria, as the prevalence of malaria parasitaemia among eligible children aged 6–59 months in the state was 25.7% in 2021, the highest in the south-east geopolitical zone and higher than the national prevalence of 22.3% [3].

Malaria could be diagnosed by the use of clinical symptoms (presumptively) and by parasitological diagnostic testing using malaria rapid diagnostic test (RDT), light microscopy, and polymerase chain reaction (PCR) [4, 5]. Following the declining malaria incidence in high burden countries, emergence of parasite resistance to anti-malarial drugs, particularly artemisinin-based combination therapy (ACT), and the increased availability of diagnostic testing with RDT [6, 7], the World Health Organization (WHO) recommended in 2010 that all patients suspected of having malaria receive prompt parasitological diagnostic testing (with microscopy or RDT) to confirm diagnosis before treatment [5]. The availability and use of RDT is a vital part of the strategy for this recommendation by the WHO [5, 6, 8, 9] because RDT is much more feasible and cheaper to deploy and use by PHC workers even in remote rural settings and has thus enhanced availability and accessibility to diagnostic testing. To make universal parasitological diagnostic testing achievable, many developing countries, including Nigeria, with the support of foreign partners, have scaled-up the availability and use of RDT particularly by primary healthcare (PHC) workers who are at the frontline in healthcare provision, especially in the rural areas [10].

Also, universal parasitological diagnosis of malaria before treatment has been recommended by the Nigerian National Guidelines for Diagnosis and Treatment of Malaria [11, 12]. In the foregoing regard, the United States Agency for International Development President's Malaria Initiative (USAID PMI) provides support or supplies RDT kits (and ACT) to many PHC facilities in Ebonyi state for free or subsidized malaria diagnostic (and treatment) services.

The problem of over-treatment of malaria with ACT and heightened risk of selection pressure and drug resistance emerged following the progressive and widespread increase in the use of ACT across Nigeria [13–15], and sub-Saharan African countries [16, 17], after ACT was recommended as the first-line anti-malarial treatment in 2006 [18]. Adherence to the recommendation of universal diagnostic testing before malaria treatment could help address this problem [5, 11, 12], however, many health workers in Nigeria still relied only on presumptive diagnosis [19–24] and many still inappropriately prescribed ACT following negative RDT results [25–27]. This in-appropriate prescription of ACT for patients with negative RDT results is perpetuating the problem of over-treatment of malaria (and the higher risk of drug resistance) despite diagnostic testing with RDT [28].

Only few qualitative studies have been conducted to provide more insights about the determinants of the use of RDT and anti-malarial drug prescription practices by health workers, particularly in the context of increased RDT availability and in Nigeria. With the use of RDT at the PHC level where another form of parasitological diagnosis of malaria, such as microscopy, has been hard to come by, universal access to parasitological diagnosis of malaria has become more feasible [4]. There was need for qualitative studies in the foregoing regards.

The aim of this qualitative study was to explore the determinants of the use of RDT and anti-malarial drug prescription practices, including appropriate response to negative RDT results, by PHC workers in Ebonyi state, Nigeria, in order to generate empirical evidence to inform relevant health policy actions.

## Methods

### Study design and participants

This qualitative study was part of a concurrent independent mixed method study among PHC workers in Ebonyi state, Nigeria. Eligible participants were those involved in the diagnosis and treatment of malaria at PHC facilities providing maternal and child healthcare services, including immunization, who had at least one year of practicing experience and gave written consent. Most of the PHC workers at the PHC facilities were community health extension workers (CHEWs) and health attendants. Nurses and midwives, community health officers (CHOs), and environmental health officers also did work at the PHC facilities. The CHEWs and CHOs are mid-level health workers in Nigeria who receive formal training to provide basic essential and public health services in PHC facilities and in the communities. Health attendants are health assistants or community resource persons (CORPs) that received informal training at PHC facilities and are allowed to treat minor ailments like uncomplicated malaria. The CHEWs comprise of senior CHEWs (SCHEWs) and junior CHEWs (JCHEWS).

Based on the investigator’s judgement, 23 eligible participants were selected purposively from the main cadres of PHC workers and across the three senatorial zones with the intention of getting rich information and diverse opinions and to enhance transferability of findings.

### Data collection and analysis

Data was collected through focus group discussions (FGDs), using pre-tested FGD guide, between March 2 and 10, 2020. The FGD guide was written in English and had seven stem questions each with probes. The seven stem questions were on: ways of diagnosing malaria; use of RDT for malaria diagnosis; how malaria was usually diagnosed; how malaria patients were treated; how malaria patients were treated following RDT results; factors that influenced the use of RDT for malaria diagnosis; and factors that influenced anti-malarial drug prescription.

The investigator administered three FGDs in English using an FGD guide and a research assistant was a note-taker. Permission was taken from the participants for the discussions to be audio-recorded. Eleven discussants (nine PHC workers, one note-taker, and the investigator) took part in one FGD while nine discussants (seven PHC workers, one note-taker, and the investigator) took part in each of the other two FGDs. No new theme emerged during the third FGD and saturation was considered achieved. Each FGD lasted for about 40 min. The audio recordings were transcribed verbatim by an experienced staff who was the note-taker during the FGDs.

The investigator did the data (transcripts) verification and analysis and interpretation and these were informed by the thematic analytic method recommended by Braun and Clarke [29]. The transcripts were compared with the audio recordings by simultaneously reading the transcripts and listening to the corresponding recordings and by re-reading transcripts and replaying corresponding recordings back-and-forth iteratively and systematically to check for “accuracy”. Familiarization with the data was done by reading and re-reading the data and taking note of initial ideas. This was followed by coding, searching for themes or patterns, reviewing themes, defining and naming themes, and producing the report [29]. The reporting of this study was guided by the Standards for Reporting Qualitative Research (SRQR) [30].

## Results

A total of 23 PHC workers participated in the FGDs including senior community health extension workers (SCHEWs), junior community health extension workers (JCHEWs), and health attendants. Females were 20 (87.0%), CHEWS (SCHEWs and JCHEWs) were 15 (65.2%) and health attendants were 8 (34.8%).

### Knowledge about malaria diagnosis and malaria rapid diagnostic test

The majority of the participants said malaria could be diagnosed via two main ways such as microscopy and RDT while some said malaria could also be diagnosed by using symptoms. The majority knew the meaning of malaria RDT and all knew how to carry out RDT. Typical quotes from the participants are presented in Table 1.

**Table 1:** Illustrative quotes for the knowledge of participants about malaria diagnosis and malaria rapid diagnostic test

“It [malaria diagnosis] has two ways: Number one is microscopic, using the microscope, another one is malaria RDT.” (Male)
“We can diagnose [malaria] using microscopy and RDT.” (Female)
“You can also diagnose [malaria] through the symptoms like fever, weakness of joints, vomiting, loss of appetite.” (Female)
“M stands for Malaria, R stands for Rapid, D stands for Diagnostic, T stands for Test.” (Male)
“Clean the finger, prick it, collect blood using pipette, drop inside blood well, add buffer according to maker’s instruction, read after 20 min, you will see two lines”. “There can be three results: positive, negative, invalid.” (Male)

### Perceptions about the use of malaria rapid diagnostic test for malaria diagnosis

Most participants said RDT was a quick, easy, and reliable way of diagnosing malaria and expressed confidence in the results. Some, however, noted that RDT could give negative results in some patients that actually had malaria. Some gave instances of patients with negative RDT results who did not recover following initial treatment with antibiotics but did so after anti-malarial drugs were given. They also said they preferred to use RDT to diagnose malaria because it was faster, easier, and readily available compared to microscopy and it was more reliable than using symptoms alone. Typical quotes from the participants are presented in Table 2.

**Table 2:** Illustrative quotes for perceptions of participants about the use of malaria rapid diagnostic test for malaria diagnosis

“RDT helps to identify whether the person has malaria or not. It is the easiest way to diagnose malaria.” (Female)
“RDT in the fastest way of diagnosing malaria but, to me, I don’t think it is 100%, because it is not all malaria that shows in the RDT.” (Female)
“Without RDT we cannot differentiate person with malaria, ordinary fever, because every fever is not malaria. RDT help identify malaria and we treat and help [the patients].” (Female)
“It is easier than microscopy because within 20 min you know the result, so simple and it is also reliable, very reliable, though some will not show accurate result.” (Female)
“I agree with what they were saying but sometimes if somebody presents with these symptoms, it [RDT] may read negative and if you treat with like [say] antibiotics, the sickness will not go and you now decide to treat with anti-malarial…, it works, even with the negative result, the sickness will go.” (Female)

### Use of malaria rapid diagnostic test for malaria diagnosis and anti-malarial drug prescription practices

All the participants said they routinely used RDT for malaria diagnosis and that they mostly used ACT to treat their patients with malaria, and the artemisinin-based combination mostly used was artemether-lumefantrine (AL). The correct dosage of AL was also described. All said they mostly prescribed ACT and antipyretics for positive RDT results and the majority said they mostly prescribed antibiotics (amoxicillin) and antipyretics for negative RDT results. Typical quotes from the participants are presented in Table 3.

**Table 3:** Illustrative quotes for the use of malaria rapid diagnostic test for malaria diagnosis and anti-malarial drug prescription practices by participants

“In this hospital [PHC centre] we use RDT routinely. 100% of fever in this hospital is being diagnosed using RDT. Once fever, we must use RDT. We have RDT available all the time unlike microscopy as we do not have lab scientist all the time.” (Female)
“We give coartem, AL [artemether-lumefantrine], it [the dose] depends on the age and weight of the patient….” (Female)
“On that day one, the first dose is as soon as possible, next dose 8 h [later]. Day two, morning and night, that is 12 h [apart] and so you do on the third day.” (Female)
“When the [RDT] result is positive and there is fever, we use antipyretics, paracetamol or any other, with AL [artemether-lumefantrine]. In negative cases, we do use mostly antibiotics.” (Female)
“If the person's test is positive, we treat using anti-malarial which is ACT [for] 3 days completely. If negative, we will now check [for] some other signs, like in children, difficulty in breathing, we may suspect pneumonia and give amoxil [amoxicillin].” (Female)

### Determinants of the use of malaria rapid diagnostic test for malaria diagnosis

The determinants of the use of RDT for malaria diagnosis by PHC workers according to the participants could be categorized as systemic, provider, client, and RDT related factors. Systemic factors were RDT availability and patient load (as some would not do RDT for their patients when the patient load was high due to lack of staff). Provider factors were the confidence in RDT and desire to make correct diagnosis, provider’s knowledge and training, and the fear to prick a patient. Client factors were the fear of needle prick which made clients to decline RDT and clients’ self-diagnosis of malaria (based on symptoms) and insistence on not receiving RDT. RDT factor was the ease of conducting and interpreting RDT.

The availability of RDT was reported as a major determinant because RDT could only be done when RDT kits were available at the PHC facilities (in comparison, microscopy could not be done because it was not available and the non-availability of RDT in some facilities made some PHC workers to routinely use presumptive diagnosis). Since RDT was viewed as being more reliable than the use of only clinical symptoms, the desire to get the diagnosis right also determined the use of RDT. When RDT kits were available, the receipt of trainings (on malaria diagnostic guidelines and importance of RDT) or the knowledge about the importance of RDT would influence PHC workers to routinely use RDT for malaria diagnosis. Another important determinant reported was patient load as it would be very difficult for PHC workers to conduct RDT for all eligible patients when patient load was high (high patient load was a very important factor because most PHC facilities had shortage of staff). Some participants reported that the fear to prick a patient and the fear of needle prick by some patients who declined the RDT also prevented some PHC workers from doing RDT. Patients who strongly believed they had malaria (based on typical symptoms) only went to PHC facilities to receive malaria treatment and not to receive RDT. Typical quotes from the participants are presented in Table 4.

**Table 4:** Illustrative quotes for the determinants of the use of malaria rapid diagnostic test for malaria diagnosis by primary healthcare workers

“RDT [is] used frequently because of its availability and there is no microscope [for microscopy]. Since the RDT is present in my facility, I use it more. I think the presence of RDT facilitates my using it.” (Female)
“The RDT is easy to carry out, … and also easy to read [interpret]” (Female)
“Factor that influence us to use RDT is, to know [the] actual [cause of] sickness, not based on signs and symptoms. Since the technology has made it possible for us to have this kit, I think it is the best option than to treat [compared to treating] somebody based on signs and symptoms.” (Female)
“Why some people don’t use RDT routinely is that: some don’t understand the benefit.[Also, RDT is] not supplied in some facilities, so they cannot use it and they have to buy and sometimes no money to buy so they use signs and symptoms to treat their patients.” (Female)
“Many reasons [for not using RDT]: (1) Stock out (2) Because they have much workload in the facility, they may give (anti-malarial drugs) without testing …. (3) Even some health care workers find it hard to prick [their patients], fear of lancet prick.” (Female)
“I agree with what she said but some [patients] will say don’t puncture [prick] my hand” (Female)
“For me, knowledge, training and this influence me to use commodity [RDT] supplied to me, this influences me to use it….” (Female)

### Determinants of anti-malarial drug prescription practices

The participants reported systemic (availability and cost of the drugs) and drug related (effectiveness and side-effects of the drugs) factors as determinants of anti-malarial drug prescription practices by PHC workers, including which anti-malarial drug they mostly prescribed. They said they mainly used ACT to treat malaria because ACT were more effective than other anti-malarial drugs and that artemether-lumefantrine (AL) was preferably used because it had less side-effects compared to artesunate-amodiaquine (AA). For PHC facilities that were supported or supplied with ACT for free or subsidized malaria services by USAID PMI, these artemisinin-based combinations were the mainstay of malaria treatment and because AL was supplied more that AA, AL was mostly used. In contrast, in the facilities not supported by PMI, the non-availability of ACT and the high cost of artemisinin-based combinations (for those that bought from the open market) negatively affect the use of ACT by those PHC workers who mostly used other cheaper or more available anti-malarial drugs.

The determinants of the drugs prescribed to patients following negative RDT results according to participants were provider and clients related factors. Provider factors included the desire to make more money or generate more revenue and limited confidence/trust in the reliability of RDT when the results were negative. Participants also reported that whenever the febrile illness was not abating after the first course of antibiotics (usually amoxicillin) following negative RDT results, many PHC workers would then prescribe anti-malarial drugs. Client factor was patients’ demand (pressure from patients) to be treated for malaria even when the results were negative. Patients who self-diagnosed themselves of having malaria (based on typical symptoms) only went to health facilities to receive malaria treatment irrespective of the outcome of RDT.

Regarding drug prescription following positive RDT results, an important determinant was provider factor such as the desire to make more money or generate more revenue which made many PHC workers to mostly give antibiotics in addition to anti-malarial drugs and antipyretics. Typical quotes from the participants are presented in Table 5.

**Table 5:** Illustrative quotes for the determinants of anti-malarial drug prescription practices by primary healthcare workers

“After doing test and drugs are not there, it will affect [my prescription]. If you charge high or costly [for ACTs] it will affect [patients affordability].” (Female)
“……..AL [artemethher-lumefantrine] particularly more effective and less side effects than AA [artesunate-amodiaquine] [so, I mostly prescribe AL].” (Female)
“We use that AL (artemether-lumefantrine) mostly as side effect is less than that of AA (artesunate-amodiaquine). That AA weakens some people.” (Female)
“… to me, I don’t think it [RDT] is 100% because it is not all malaria that shows in the RDT.” (Female)
“… if somebody presents with these symptoms, it [RDT] may read negative and if you treat with like [say] antibiotics, the sickness will not go and you now decide to treat with anti-malarial…, even with the negative result, the sickness will go.” (Female)
“One of the reason that make health workers give [anti-malarial drug for negative RDT results] is to please some clients, in order to retain them, meet up their demand.” (Female)
“[Prescribing] Malaria drug when [RDT is] negative: (1) Patient pressure (2) Get money from patient, something like that, but it is a wrong practice.” (Female)
“To some of the health workers, the villagers have known that the anti-malarial drug [ACT] is free, if you give them only ACT [for positive RDT], they won’t pay. If you add antibiotics, they can now pay. Ideally it is not good.” (Female)

## Discussion

This study explored the knowledge and perceptions about RDT and the determinants of the use of RDT for malaria diagnosis and anti-malarial drug prescription practices by PHC workers in Ebonyi state, Nigeria. Most of the study participants expressed good knowledge of malaria diagnosis and RDT, perceived RDT as a reliable way of diagnosing malaria, and said that they had confidence or trust in the results. Some, however, noted that RDT could give negative results in some patients that actually had malaria. All reported that they routinely used RDT for malaria diagnosis and said they preferred to use RDT for malaria diagnosis because it was faster, easier, and readily available compared to microscopy and it was more reliable than using symptoms alone.

Similarly, in a study across six states (in five geopolitical zones) in Nigeria [26], the PHC workers showed positive perceptions to the use of RDT for malaria diagnosis. Also, the health professionals in a study in Burkina Faso [31] expressed positive views about the use of RDT for malaria diagnosis and most of the health workers in a study in Uganda [32] reported that they had confidence or trust in the use of RDT for malaria diagnosis. In another study in Kenya [33], the health workers generally viewed RDT as an effective tool for malaria diagnosis but at the same time expressed some doubts about the reliability of RDT due to what they perceived as a surprisingly high rate of negative results (compared to clinical diagnosis).

The above evidence has important implications. Most participants had positive perceptions about the use of RDT for malaria diagnosis and these perceptions were enhanced by the fact that RDT was faster, easier, and readily available (compared to microscopy) and more reliable than using symptoms alone. However, the distrust for negative RDT results was still common and could limit the extent to which the problem of over-treatment of malaria with ACT and heightened risk of selection pressure and drug resistance could be prevented even if universal diagnostic testing before malaria treatment were to be achieved. Anecdotal evidence (as observed by the researcher) indicate that this lack of confidence in negative RDT results is very common among not only PHC workers but also the general population of health workers (including those at tertiary health facilities) and seems to be related to the lack of capacity to diagnose or routinely diagnose other causes of fever. In another study in Ebonyi state [34], most (79.5%) of the medical doctors (across tertiary and secondary health facilities) did not agree (strongly disagreed or disagreed or were undecided) that patients with negative RDT results should not be given anti-malarial drugs (the proportion was as high as 57.1% even with regards to microscopy). It has also been reported that lack of the ability to diagnose non-malaria fevers influenced health workers’ decision to prescribe anti-malarial drugs for patients with negative RDT results in Uganda [32, 35]. More policy actions and interventions are needed to improve health workers confidence and trust in negative RDT results.

The study participants were of the view that the determinants of the use of RDT for malaria diagnosis by PHC workers included RDT availability, the ease of conducting and interpreting RDT, confidence in RDT and the desire to make correct diagnosis, health worker’s knowledge and training, patient load, the fear by some to prick a patient, the fear of needle prick which made some patients to decline to receive RDT, and patients’ self-diagnosis of malaria (based on symptoms) and insistence on not receiving RDT. Similarly, a study across south-eastern Nigeria [36] reported that non-availability of RDT kits and doubts about their reliability enhanced the non-use of RDT (and microscopy) for malaria diagnosis. Also, the health professionals in a study in Burkina Faso [31] reported that doubts regarding the reliability of RDT results and the occasional stock-outs of RDTs kits were the reasons they predominantly used presumptive diagnosis and a study in Kenya [33] reported that availability of RDT kits, health workers’ perceptions about RDT, and patients’ expectations or demand were factors that influenced the use of RDT for malaria diagnosis.

From the perspectives of the study participants, the determinants of the prescription of anti-malarial drugs by PHC workers following negative RDT results were patients’ demand (pressure from patients), the desire to make more money or generate more revenue, and limited confidence or trust in the reliability of negative RDT results. Similarly, other studies in Kenya [33] and Uganda [32, 35] reported that patients’ or caregivers’ demand influenced the prescription of anti-malarial drugs to patients with negative RDT results as patients had expectations of being treated for malaria regardless of diagnostic test results. Also, in another study among health workers in Ghana [37], strong clinical suspicion, doubt in the accuracy of malaria test results, age of patients, and the preference of patients were reported as factors that influenced the prescription of anti-malarial drugs to patients with negative malaria tests results.

The above findings imply that when RDT results were negative, it was perhaps relatively easy for patients’ demand and pressure to influence the treatment decision of many of the PHC workers who already had the desire to make more money or revenue and who also had little or no confidence in negative RDT results. Another important factor identified during this study was the fact that when febrile symptoms were not abating following the initial course of antibiotics, usually amoxicillin, prescription for negative results, many PHC workers would immediately prescribe anti-malarial drugs. This was without considerations for the possibility that the febrile illness was due to amoxicillin-resistant bacterial infection or other non-bacterial causes. Because of limited capacity of the PHC workers, as has also been reported in Uganda [32, 35], such considerations would most likely not lead to the prescription of other more potent antibiotics or proper investigation and management of non-malarial causes of fever. However, it might at least engender early decision to refer the patient. These issues can be explored by further studies.

This study also found that the desire to make more money or revenue influenced the unnecessary co-prescription of antibiotics for patients with positive RDT results (in addition to anti-malarial drugs and antipyretics). Perhaps this finding was because in the USAID PMI supported health facilities, the ACT were given free of charge to patients once the malaria diagnostic test was positive, but the antibiotics were not. Perhaps another contributing factor could be expectations or pressure from the local government authorities for the PHC facilities to increase their internally generated revenues (as have been observed anecdotally).

The foregoing discourse highlights important systemic, provider, client, and RDT/drug related determinants of the use of RDT and anti-malarial drug prescription practices by PHC workers and thereby emphasizes the complexity of the issues that need to be addressed and the need for system-wide, supply-side and demand-side interventions in the drive to achieve universal parasitological diagnostic testing before malaria treatment with good and appropriate anti-malarial drug prescription practices by health workers in Ebonyi state, Nigeria, and similar settings. Although some of the determinants of the use of RDT and anti-malarial drug prescription practices identified by this study corroborate those of previous studies, others are perhaps added evidence including: the ease of conducting and interpreting RDT, the desire to make correct diagnosis, patient load, fear to prick a patient, and clients’ fear of needle prick (and objection to RDT) as determinants of the use of RDT for malaria diagnosis; drug availability, cost, effectiveness, and side-effects as determinants of anti-malarial drug prescription practices; and the desire to make more money or revenue as a determinant of the prescription of anti-malarial drugs following negative RDT results and of the unnecessary co-prescription of antibiotics with anti-malarial drugs following positive RDT results. Subsequent supply-side and demand-side interventional studies on the effects of some of the above unique determinants on the use of RDT and anti-malarial drug prescription practices by PHC workers are imperative.

As a limitation, this study was subject to reporting bias as there was the tendency for participants to overstate desirable perceptions or practices and or understate (avoid stating) undesirable perceptions or practices. The identification of determinants was based on the subjective perceptions of participants which could be biased by personal sentiments and interests. However, measures were taken to minimize such bias, overall, by selecting different categories of participants to give diversity of views, asking more general and indirect questions as much as possible, and by assuring participants of and ensuring high degree of confidentiality.

## Conclusions

This study has shown that there were many systemic, provider, client, and RDT/drug related determinants of PHC workers’ use of RDT and anti-malarial drug prescription practices, including appropriate response to negative RDT results, that should provide tailored guidance for relevant health policy actions in Ebonyi state, Nigeria, and similar settings.

## Data Availability

The empirical data underlying the analytic findings are appropriately presented in the manuscript text as quotes. Because of the sensitive and descriptive nature of the underlying data, further empirical data (including anonymized data) will not be made available to protect participants’ confidentiality.
